# Circulating IGF-binding protein 7 (IGFBP7) levels are elevated in patients with endometriosis or undergoing diabetic hemodialysis

**DOI:** 10.1186/1477-7827-6-54

**Published:** 2008-11-19

**Authors:** Masahiko Kutsukake, Ryosuke Ishihara, Katsutoshi Momose, Keiichi Isaka, Osamu Itokazu, Chinatsu Higuma, Takeshi Matsutani, Akihisa Matsuda, Koji Sasajima, Takahiko Hara, Kazuhiro Tamura

**Affiliations:** 1 Department of Endocrine Pharmacology, Tokyo University of Pharmacy and Life Sciences, 1432-1, Horinouchi, Hachioji, Tokyo 193-0392, Japan; 2 Department of Obstetrics and Gynecology, Tokyo Medical University, 6-7-1 Nishishinjuku, Shinjuku-ku, Tokyo 160-0023, Japan; 3 Surgery for Organ Function and Biological Regulation, Graduate School of Medicine, Nippon Medical School, 1-7-1 Nagayama, Tama, Tokyo 206-8512, Japan; 4 Stem Cell Project Group, the Tokyo Metropolitan Institute of Medical Science, 3-18-22 Honkomagome, Bunkyo-ku, Tokyo 113-8613, Japan

## Abstract

**Background:**

Insulin-like growth factor-binding protein-7 (IGFBP7) is a secretory protein with a molecular mass of approximately 30 kDa. It is abundantly expressed in the uterine endometrium during the secretory phase of the menstrual cycle. Decreased IGFBP7 expression has been observed in some cancers and leiomyomata.

**Methods:**

To determine whether serum IGFBP7 levels reflect changes in uterine IGFBP7 expression in humans during the menstrual cycle, and to examine whether serum IGFBP7 levels are altered in patients with various disorders, we developed a novel, dual-antibody sandwich enzyme-linked immunosorbent assay (ELISA). Firstly, concentrations of IGFBP7 released into the medium were determined in cultured endometrial stromal and glandular cells. Blood samples were collected from women who had normal menstrual cycles and who had been diagnosed with endometriosis. Serum from hemodialysis patients and gastrointestinal cancers was also used to determine the IGFBP7 levels.

**Results:**

Using this new ELISA, we demonstrated that cultured uterine cells secrete IGFBP7 into the medium. Patients with endometriosis and those with type II diabetes mellitus undergoing hemodialysis had significantly higher serum concentrations of IGFBP7 than the relevant control subjects. There were no differences in serum IGFBP7 levels in women at different stages of the menstrual cycle. Furthermore, serum IGFBP7 levels in patients with colorectal, esophageal, or endometrial cancer were not different than normal healthy subjects.

**Conclusion:**

Our observations suggest that IGFBP7 is associated with the pathophysiology of endometriosis and diabetes mellitus, and that serum IGFBP7 levels do not reflect enhanced uterine expression of IGFBP7 mRNA during the menstrual cycle.

## Background

We are interested in factors that play a crucial role in the establishment of pregnancy. Previously, we found that IGFBP7 is highly expressed in the rat uterus during implantation [1]. IGFBP7, also known as IGFBP-related protein 1 (IGFBP-rP1), mac25, prostacyclin stimulating factor (PSF), and angiomodulin [2], is a secreted glycoprotein of the IGFBP-rP family, which binds insulin and insulin insulin-like growth factor I (IGF-I). However, unlike IGFBPs 1–6, IGFBP7 has a relatively low affinity for IGF, while its affinity for insulin is 500-fold higher [3]. At present, the biological functions of IGFBP7 are unclear. While it is expressed in a wide variety of human tissues, including gut [4], it is notably abundant in female reproductive organs such as the mature follicle [5], the corpus luteum [6], and the uterus [7,8]. IGFBP7 is present in uterine glandular epithelial cells and stromal cells, particularly during the secretory phase of the menstrual cycle, and IGFBP7 mRNA levels in late secretory stage are approximately 150-fold higher than in the proliferative stage [7]. Finally, uterine leiomyomata express lower levels of IGFBP7 than the adjacent myometrium [9].

High grade invasive breast cancers lack IGFBP7 expression [10,11], which suggests that IGFBP7 may be associated with tumorigenesis. Patients with low IGFBP7 expression levels had reduced survival as compared to patients with higher expression levels [12]. In fact, the gene encoding IGFBP7 was originally identified on the basis of its reduced mRNA expression levels in meningioma cell lines relative to normal cells [13]. In addition, recombinant IGFBP7 suppresses the growth of cultured human tumor cells through a mechanism that involves an increase in the number of cells in the G1 phase of the cell cycle [14].

Here, we report the development of an ELISA for measuring serum IGFBP7. Our objective was to test the hypothesis that circulating IGFBP7 levels reflect localized changes IGFBP7 expression, particularly in the uterus, in which there is a marked increase in IGFBP7 expression during the secretory phase of the menstrual cycle, and in solid tumors, in which there is reduced expression of IGFBP7. We examined whether changes in uterine IGFBP7 expression during the menstrual cycle correlated with serum IGFBP7 levels. Given the association between IGFBP7 and disease, we also compared serum IGFBP7 levels in patients with endometriosis or endometrial, colorectal or esophageal cancer to normal healthy individuals. Lastly, we examined serum IGFBP7 levels in patients undergoing hemodialysis, many of whom had type II diabetes mellitus. We found that circulating levels of IGFBP7 are enhanced in patients with endometriosis and type II diabetes. However, peripheral blood levels of IGFBP7 did not correlate with increased IGFBP7 expression in the uterus during the secretory phase of the menstrual cycle.

## Methods

### Preparation of subconfluent uterine cells and blood collection

Endometrial stromal cells (ESCs) from the proliferative phase of the menstrual cycle were prepared as described previously [8]. ESCs were determined to be at the proliferative phase based on the patients' menstrual history, serum estradiol and progesterone levels at the time of sample collection, and histological examination using standard histological criteria. The samples were collected after obtaining informed consent from the patients in accordance with the Helsinki Declaration and the requirements of the Clinical Research Ethics Committee of the Tokyo Medical University Hospital (Shinjuku, Tokyo). Cells were cultured in DMEM/F-12 medium (Invitrogen, Carlsbad, CA, USA) containing 10% (v/v) charcoal-dextran-treated fetal bovine serum (10% stripped FBS, Hyclone, South Logan, UT, USA) and antibiotics (50 μg/ml penicillin, 50 μg/ml streptomycin, 100 μg/ml neomycin, 100 μg/ml gentamicin and 2.5 μg/ml fungizone, Invitrogen). Subconfluent cells were cultured in 12-well culture plates with DMEM/F-12 medium containing 2% (v/v) stripped FBS and antibiotics. The IGFBP7-specific siRNA (Darmacon Inc., Chicago, IL, USA) was used as previously described [8]. The glandular cell line EM1, which was originally called EM-E6/E7/TERT-1(EM-1) [15], was cultured in DMEM containing 10% (v/v) FBS (JRH Biosciences, ACSL, Lenexa, MD) and antibiotics, as described above. Blood samples were collected from 62 women between the ages of 23 and 45 who had normal menstrual cycles. The menstrual cycle stage of each subject was determined based on menstrual history and serum estradiol and progesterone levels at the time of sample collection. Blood samples were obtained from 11 women who had been diagnosed with endometriosis, 33 men undergoing hemodialysis, and 58 men requiring elective open surgery for colorectal (n = 40) or esophageal cancers (n = 18). Of the hemodialysis patients, 18 had been diagnosed with type II diabetes mellitus. This study was performed according to the Helsinki Declaration and was approved by the Ethical Committee of Nippon Medical School Hospital. Signed informed consent to participate in the study was obtained from all patients prior to phlebotomy or surgery.

### IGFBP7 ELISA

IGFBP7 levels in culture media and serum were measured using a novel sandwich ELISA, which incorporated a polyclonal and a monoclonal anti-IGFBP7 antibody (R&D Systems Inc.). The polyclonal anti-IGFBP7 antibody was diluted to 1 μg/ml in PBS and incubated overnight at room temperature (R/T) in poly-L-lysine-coated 96-well microtiter plates. The wells were washed with PBS containing 0.1% Tween-20 (PBST), blocked with 3% (w/v) BSA in PBS at R/T for 1 hour (h), and then washed again with PBST, after which samples diluted with PBS containing 0.1% (w/v) BSA(PBSB) were added. Recombinant human IGFBP7 (R&D Systems Inc.) at concentrations of 0.24, 0.97, 3.9, 15.6, or 62.5 ng/ml in PBSB was also added as a standard. Plates were incubated for 2 h at R/T, and then the wells were washed with PBST. Monoclonal anti-IGFBP7 antibody (1 μg/ml) in PBSB was then added. After another period of incubation for 2 h at R/T, the plates were washed with PBST, incubated for 1 h at R/T with 1 μg/ml peroxidase-conjugated goat anti-mouse IgG antibody (Vector Lab. Inc.), and then washed again with PBST. A substrate reagent (100 μl) (R&D Systems Inc.) was added to the wells, and the chromogenic reaction was allowed to proceed for 10 minutes at R/T, at which point 50 μl of 2 N HCl was added to each well to stop the reaction. The plate was read at 450 nm in a microtiter plate reader (SAFIRE, Wako Pure Chemical, Osaka, Japan). Inter- and intra-assay coefficients of variation were 19.9% and 14.0%, respectively. The average percent recovery was approximately 100%. The sensitivity of the assay was 0.3 ng/mL.

### Statistics

Results are presented as means ± standard error (SE), and differences were analyzed using the Student t test or ANOVA test. A P value of less than 0.05 was considered to be statistically significant.

## Results

**Figure 1:**
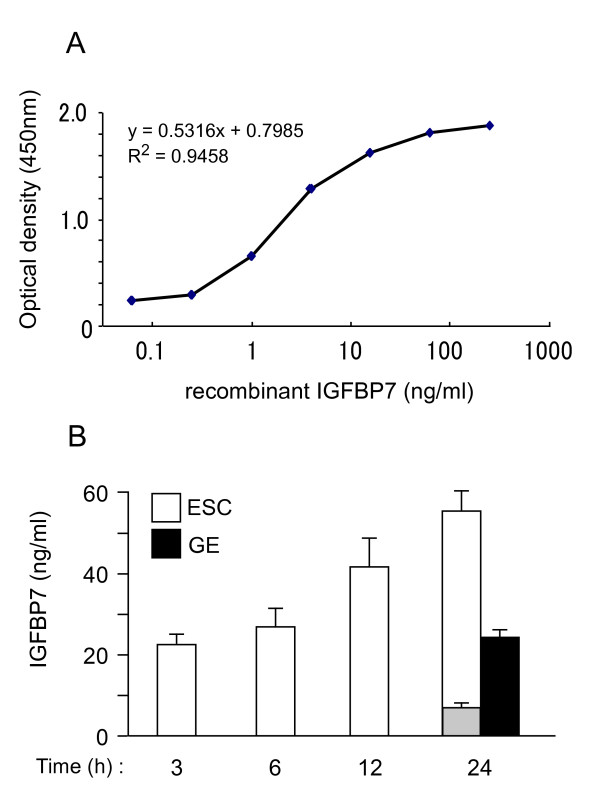
**Determination of IGFBP7 protein levels in conditioned media from human ESCs and GE cells**. The conditioned media of subconfluent cells was collected at the indicated times and stored frozen until use. (A) Representative standard curve obtained by ELISA using recombinant human IGFBP7 in PBS containing 0.1% BSA. Data represents the means of four replicates. (B) IGFBP7 levels in conditioned media over time. The conditioned medium of GE cells was measured at the 24 h time point only. Gray column; IGFBP7 levels in the medium of ESCs treated for 24 h with IGFBP7 siRNA (10 pmol). The data represents the means ± SE of three separate cultures, assayed in duplicate.

We developed a novel IGFBP7 ELISA using two anti-IGFBP7 antibodies, as described in Materials and Methods. The standard curve generated with recombinant IGFBP7 was linear between 0.3 and 20 ng/ml (Fig. 1A). We recently demonstrated that IGFBP7 is expressed in the human uterus, and that IGFBP7 mRNA levels are up-regulated in ESCs and glandular epithelial cells during the receptive phase of the menstrual cycle [8]. We first determined whether cultured primary ESCs and EM1 glandular epithelial cells (GE cells) secreted IGFBP7. IGFBP7 gradually accumulated in the culture medium of ESCs and GE cells over a 24 h period of incubation, reaching levels of 55 and 25 ng/ml, respectively, at 24 h (Fig. 1B). When ESCs were treated with IGFBP7 siRNA to knock-down the expression of IGFBP7, the protein levels of IGFBP7 were reduced by 86% (approximately 7.5 ng/mL). These results confirmed that cultured ESCs secrete IGFBP7, and demonstrated that ESCs secrete about 2-fold more IGFBP7 than GE cells.

**Figure 2:**
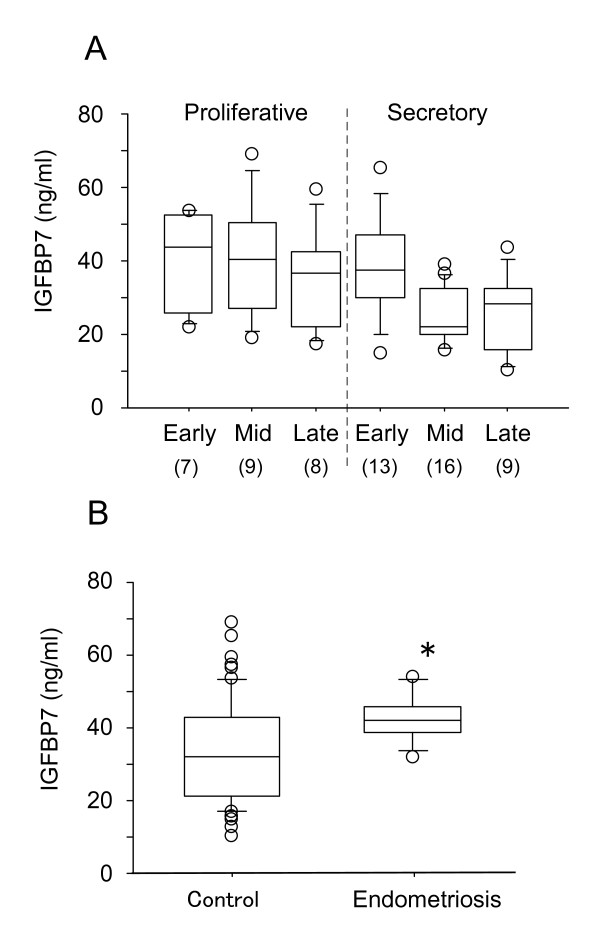
**Serum IGFBP7 levels in patients at different phases of the menstrual cycle (A) and with endometriosis (B)**. (A) The sera from 62 women with regular menstrual cycles were tested. The numbers in parentheses indicate the number of subjects at each stage. (B) The sera from 11 patients with endometriosis were tested along with those of the 62 women with regular menstrual cycles, described for A, who served as controls for this study (Control). Data represents the means ± SE. *P < 0.05 as compared to Control.

There are marked changes in uterine IGFBP7 mRNA levels during the menstrual cycle. To determine if these changes are reflected in serum IGFBP7 levels, we measured IGFBP7 concentrations in sera from 62 women at different phases of the menstrual cycle (Fig. 2A). Serum IGFBP7 levels did not change significantly throughout the menstrual cycle. The average IGFBP7 level in serum was 33 ± 13.1 ng/ml (median of 35 ng/ml). We also observed that mean serum IGFBP7 levels do not differ between males and females (data not shown).

**Figure 3:**
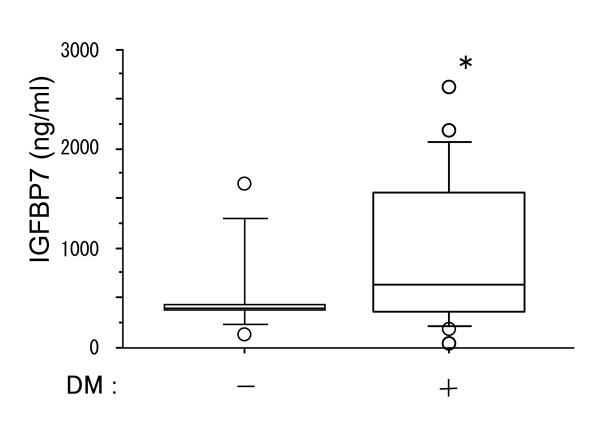
**Serum IGFBP7 levels in hemodialysis patients with or without type II diabetes (DM)**. Serum from 33 hemodialysis patients without DM (15 subjects) and with DM (18 patients) was collected. *P < 0.05 as compared to without DM (Control).

**Figure 4:**
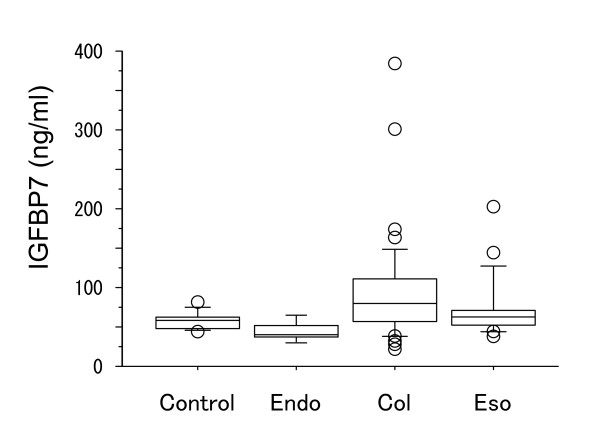
**Serum IGFBP7 levels in patients with endometrial cancer (Endo), colon cancer (Col) or esophageal cancer (Eso)**. Serum was collected from each patient (5 Endo, 40 Col, 18 Eso) before operation.

Analysis of serum IGFBP7 levels in patients with endometriosis revealed that these patients had significantly higher average levels of IGFBP7 than control subjects (Fig. 2B). For this study, the 62 female subjects with normal menstrual cycles that were analyzed in Fig. 1 served as control subjects. Based on a previous report by López-Bermejo et al. [16] showing an association of serum IGFBP7 with insulin resistance, we examined the levels of IGFBP7 in non-diabetic and diabetic patients receiving hemodialysis. Patients with type II diabetes mellitus (DM) had significantly higher IGFBP7 levels than non-DM patients (Fig. 3). Analysis of serum IGFBP7 levels of patients with endometrial, colon or esophageal cancer revealed that IGFBP7 levels did not differ significantly from those of control subjects (Fig. 4).

## Discussion

Endometriosis, which is defined as the presence and proliferation of endometrial glands and stroma outside the uterine cavity, is one of the most common benign gynecological diseases in women of reproductive age [17]. The disease is estrogen dependent and is most often treated using gonadotropin-releasing hormone (GnRH) agonists, with the aim of downregulating ovarian estrogen production. We found that women with endometriosis had significantly higher serum IGFBP7 levels than normal women. The pathophysiological mechanism underlying endometriosis remains unclear but may involve a dysfunction of the immune system. It is believed that during the development of endometriosis, immune cells (mainly macrophages) are recruited into the peritoneal cavity, where they engage in phagocytosis of retrograde endometrial debris [18]. Peritoneal macrophages contribute to the progression of endometriosis by producing a variety of soluble inflammatory mediators [19,20]. Supporting this scenario is that subjects with endometriosis have significantly higher serum levels of interleukin (IL)-6, monocyte chemotactic protein-1, and interferon-gamma as compared to control women [21]. Significantly, IL-6 has been shown to be a possible nonsurgical marker for predicting the development of endometriosis [22]. IGFBP7 expression has been shown to be up-regulated in prostate cells by transforming growth factor (TGF)-β, IGF-I, and retinoic acid [23], and in osteoblasts by cortisol [24]. It is possible that the increased serum IGFBP7 levels seen in patients with endometriosis reflect inflammation in the pelvic cavity and the production of various cytokines, which can elevate IGFBP7 production.

Various lines of evidence suggest that IGFBP7 inhibits tumor progression [10-14,25]. Immunohistochemical analysis revealed that while normal breast tissues show strong IGFBP7 expression, invasive breast cancer tissues do not express IGFBP7 [26]. On the contrary, IGFBP7 has also been identified as a gene that is up-regulated in inflammatory breast cancer (IBC) [27]. IBC is a unique type of invasive breast cancer which progresses aggressively with the extensive erythema and edema, and dimpling of the skin. Up-regulation of IGFBP7 in IBC seems to contradict the above reports [10-14,25], which is that IGFBP7 inhibits tumor progression. Although the molecular association of IGFBP7 with tumorigenesis are still unknown, this factor might be related to angiogenesis and/or inflammation, because IGFBP7 is an endothelial marker that is expressed at higher levels in the vasculature of malignant tissues than in normal endothelial cells [28].

There is some suggestion that the gene for IGFBP7 may be silenced by promoter hypermethylation. Regional DNA hypermethylation of CpG islands commonly promotes tumor progression by silencing the expression of tumor suppressor genes [29]. Komatsu et al. [30] found an inverse relationship between the degree of DNA methylation associated with hepatocarcinogenesis and the expression levels of IGFBP7. However, despite these strong links between IGFBP7 and tumorigenesis, we did not observe a relationship between serum IGFBP7 and three different types of cancer. It is possible that local changes in IGFBP7 are not reflected in IGFBP7 levels in the peripheral blood, at least with regard to cancer. Previously, analysis using immunohistochemical staining [4] revealed intense IGFBP7-positive staining in the epithelia of distal tubules of the breast, bronchus, and kidney, and peripheral nerve fibers and glial cells of the cerebellum. The adrenal gland, with the exception of the area of zona fasciculate, also exhibited intense staining. Most endothelial cells were positive for IGFBP7, whereas fat cells, plasma cells, and lymphocytes were negative. Thus, IGFBP7 appears to be ubiquitously expressed in many organs, and could potentially be released from many sources into circulation. This may be one of the reasons why we could not detect local changes in IGFBP7 by measuring serum levels of IGFBP7. Because IGFBP7 binds to the extracellular matrix (ECM) components type IV collagen and heparin sulfate proteoglycan [2,3], most uterine IGFBP7 might be trapped in the ECM surrounding the endometrial cells. In addition, we previously demonstrated that intracellular IGFBP7, not secreted IGFBP7, is important for uterine decidualization [8]. Since high level expression of IGFBP7 was also found in blood vessels of the endometrium [8], IGFBP7 might effect vascular function in the endometrium. Thus, changes in IGFBP7 expression associated with uterine physiology might not be reflected in serum levels. Identifying the potential source of changes in serum IGFBP7 under pathological conditions might prove to be difficult.

Hemodialysis patients with diabetes had significantly higher serum IGFBP7 levels than hemodialysis patients without diabetes. This result seems to be partially consistent with a previous report [16], in which circulating IGFBP7 levels in non-diabetic men correlated negatively with serum adiponectin levels, and positively with serum C-reactive protein (CRP) and soluble tumor necrosis factor receptor 2 (sTNFR2) levels, indicating that IGFBP7 is associated with insulin resistance. IGFBP7 inhibits the binding of insulin to its receptor and decreases tyrosine phosphorylation of the insulin receptor and insulin-receptor substrate I [31]. Serum levels of IGFBP7 were clearly higher in both non-diabetic and diabetic hemodialysis patients as compared to other conditions. While our results do not provide an explanation for this for this difference, it is possible that hemodialysis triggers IGFBP7 release. However, since insulin resistance is also evident in non-diabetic dialysis patients [32], the increase in serum IGFBP7 levels might also be due to insulin resistance. Regardless of the mechanism, the results of the current study support the idea that IGFBP7 is associated with insulin resistance in non-insulin dependent diabetes mellitus patients.

## Competing interests

The authors declare that they have no competing interests.

## Authors' contributions

MK, RI, KM, TH establishment of new Elisa assay and the collection of all data (IGFBP7 levels in culture media and sera) and discussion. KI, OI, CH the collection of blood from the patients in department of obstetrics & gynecology, and discussion. TM, AM, KS the collection of blood from the patients in department of surgery of digestive organ, and discussion. KT acquisition of research grant, planning of this study and manuscript preparation. KT confirmed that the authors read and approved the final manuscript.
